# Impact of COVID-19 Pandemic on Global Poliovirus Surveillance

**DOI:** 10.15585/mmwr.mm695152a4

**Published:** 2021-01-01

**Authors:** Delayo J. Zomahoun, Ashley L. Burman, Cynthia J. Snider, Claire Chauvin, Tracie Gardner, Jacquelyn S. Lickness, Jamal A. Ahmed, Ousmane Diop, Sue Gerber, Abhijeet Anand

**Affiliations:** ^1^Polio Eradication Department, World Health Organization, Geneva, Switzerland; ^2^Global Immunization Division, Center for Global Health, CDC; ^3^Bill and Melinda Gates Foundation, Seattle, Washington.

On January 30, 2020, the World Health Organization (WHO) declared coronavirus disease 2019 (COVID-19) a Public Health Emergency of International Concern (*1*). On March 24, 2020, the Global Polio Eradication Initiative (GPEI) suspended all polio supplementary immunization activities and recommended the continuation of polio surveillance (*2*). In April 2020, GPEI shared revised polio surveillance guidelines in the context of the COVID-19 pandemic, which focused on reducing the risk for transmission of SARS-CoV-2, the virus that causes COVID-19, to health care workers and communities by modifying activities that required person-to-person contact, improving hand hygiene and personal protective equipment use practices, and overcoming challenges related to movement restrictions, while continuing essential polio surveillance functions (*3*). GPEI assessed the impact of the COVID-19 pandemic on polio surveillance by comparing data from January to September 2019 to the same period in 2020. Globally, the number of acute flaccid paralysis (AFP) cases reported declined 33% and the mean number of days between the second stool collected and receipt by the laboratory increased by 70%. Continued analysis of AFP case reporting and stool collection is critical to ensure timely detection and response to interruptions of polio surveillance.

The primary means of detecting poliovirus circulation is through syndromic surveillance* for AFP among children aged <15 years by testing stool specimens for laboratory confirmation of poliovirus.^†^ In many locations, environmental surveillance supplements AFP surveillance through the regular collection and testing of sewage to assess the geographic distribution and duration of poliovirus circulation. AFP stool specimens and sewage samples are tested in WHO-accredited laboratories within the Global Polio Laboratory Network (GPLN).^§^ This report describes the impact of the COVID-19 pandemic on polio surveillance by comparing polio surveillance data (i.e., the numbers of AFP cases reported, AFP cases with two stool specimens collected, active environmental sites collecting specimens, specimen transportation time to laboratories, and specimens tested) during the first 9 months of 2019 with those during the same period in 2020, using data reported to GPEI’s Polio Information System (POLIS).^¶^ Following the declaration of the COVID-19 pandemic a Public Health Emergency of International Concern, GPEI created a dashboard using POLIS data to flag country-level changes in the WHO Africa Region (AFR), the Region of the Americas (AMR), Eastern Mediterranean Region (EMR), European Region (EUR), South-East Asia Region (SEAR), and the Western Pacific Region (WPR). The dashboard was used to compare the number of reported AFP cases in 2019 with the number reported during 2020, as well as the collection and testing of laboratory specimens from AFP cases and environmental sites, to identify changes in surveillance before and during the pandemic (*3*). In addition, data from a separate reporting mechanism that GPEI developed to track delays in specimen transport and testing in WHO-accredited laboratories within the GPLN were reviewed for changes to routine laboratory activities and availability of resources.

## Acute Flaccid Paralysis Surveillance

**FIGURE 1:**
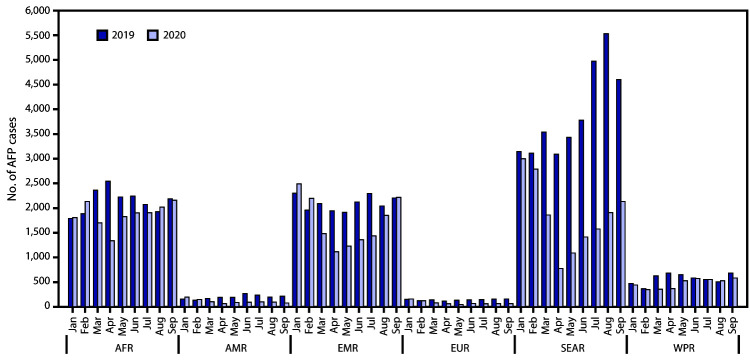
Monthly reported acute flaccid paralysis (AFP) cases, by World Health Organization region, worldwide — 2019 and 2020 **Abbreviations: **AFR = African Region; AMR = Region of the Americas; EMR = Eastern Mediterranean Region; EUR = European Region; SEAR = South-East Asia Region; WPR = Western Pacific Region.

Worldwide, the number of AFP cases reported during January–September declined 33%, from 81,439 in 2019 to 54,631 in 2020. The decline in reported AFP cases from 2019 to 2020 varied by region, with the largest decline in SEAR (53%), followed by AMR (45%), EUR (43%), WPR (20%), EMR (19%), and AFR (13%) (Table). The difference in monthly reported AFP cases in 2020 compared with those in 2019 varied widely across all regions (Figure 1). Among 159 countries for which data were available, AFP case reporting increased from 2019 to 2020 in 29 countries, most notably in Burkina Faso (292 to 864; 196%), Côte D’Ivoire (324 to 523; 61%), Zambia (157 to 228; 45%), Guinea (182 to 264; 45%), and the Philippines (552 to 726; 32%). Declines in reported AFP cases were observed in 122 countries and were largest in Indonesia (1,416 to 316; 78%), Papua New Guinea (194 to 55, 72%), Congo (161 to 72, 55%), India (31,539 to 14,842, 53%), Niger (721 to 414; 43%), and Pakistan (11,070 to 8,863; 20%). Pakistan is one of two countries with ongoing wild poliovirus circulation. No change in AFP case reporting was noted in eight countries.

**TABLE:** Polio surveillance system data reported during COVID-19 pandemic — worldwide and by region, January–September 2019 and 2020

Characteristic	Region						
	AFR	AMR	EMR	EUR	SEAR	WPR	Global
**AFP surveillance**							
**No. of AFP cases reported**							
2019	19,227	1,766	18,860	1,279	35,176	5,130	81,438
2020	16,778	967	15,359	728	16,526	4,273	54,631
% Change 2019–2020	–13	–45	–19	–43	–53	–20	–33
**% of AFP cases with two stool specimens collected**							
2019	99.2	—*	97.1	93.9	97.4	94.2	95.4
2020	99.2	—	97.2	94.1	94.8	94.4	95.1
% Change 2019–2020	none	—	0.1	0.2	–3	0.2	–0.3
**No. of days from paralysis onset to 2nd stool collection (mean)** ^†^							
2019	10	—	8.1	7.2	8.7	10.3	9
2020	9.9	—	8.3	7.9	9.8	9.7	9.4
% Change 2019–2020	–1	—	2	10	13	–6	4
**No. of days from second stool collection to receipt in lab**							
**Mean**							
2019	7.9	—	4.6	—	3.8	—	5.4
2020	11.6	—	6.2	—	11.3	—	9.2
% Change 2019–2020	47	—	13	—	197	—	70
**Median**							
2019	4	—	3	—	3	—	3
2020	7	—	4	—	4	—	4
% Change 2019–2020	75	—	33	—	33	—	33
**Environmental surveillance** ^§^							
**No. of samples per environmental surveillance site per month (mean)**							
2019	1.6	—	1.1	—	2.1	—	1.6
2020	1	—	1	—	1.6	—	1.1
% Change 2019–2020	–38	—	–9	—	–24	—	–31
**Laboratory surveillance**							
**No. of human specimens tested**							
2019	44,366	1,513	42,816	7,568	69,288	1,505	167,056
2020	37,625	848	34,597	3,038	29,699	2,892	108,699
% Change 2019–2020	–15	–44	–19	–60	–57	92	–35
**No. of environmental samples tested**							
2019	4,724	—	1,741	2,762	1,599	408	11,234
2020	2,968	—	1,630	1,713	1,103	439	7,853
% Change 2019–2020	–37	—	–6	–38	–31	8	–30

**Abbreviations:** AFP = acute flaccid paralysis; AFR = Africa Region; AMR = Region of the Americas; COVID-19 = coronavirus disease 2019; EMR = Eastern Mediterranean Region; EUR = European Region; SEAR = South-East Asia Region; WPR = Western Pacific Region.

* Data not available.

^†^ 2019 = 2,718, 2020 = 1,950 cases with no second stool specimen collected and not included in flag calculation.

^§ ^Environmental site details for EUR and WPR are incomplete.

**FIGURE 2:**
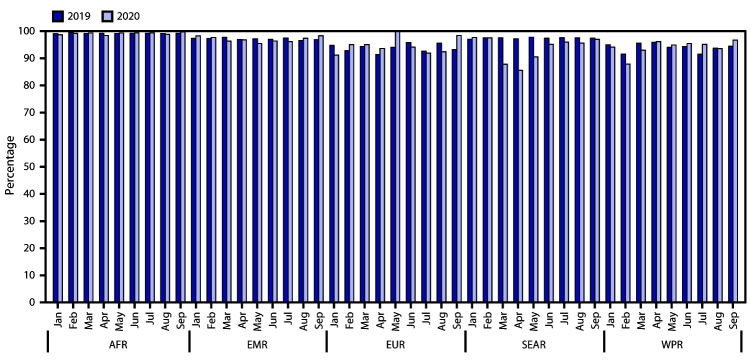
Percentage of acute flaccid paralysis cases with two stool specimens collected, by World Health Organization (WHO) region — five WHO regions,* 2019 and 2020 **Abbreviations: **AFR = African Region; EMR = Eastern Mediterranean Region; EUR = European Region; SEAR = South-East Asia Region; WPR = Western Pacific Region. * Data for the number of cases with two stools are not available for the Region of the Americas.

Overall, the percentage of AFP cases with two stool specimens collected declined 0.3% (from 95.4% in 2019 to 95.1% in 2020). A monthly comparison across the regions for January–September 2020 found that the collection of two stools ranged from a low of 85.5% in SEAR in April to a high of 100% in EUR in May (Figure 2). The percentage of AFP cases with two stool specimens fluctuated monthly during 2020, with an observed 1.3% difference from the lowest to the highest reported in AFR, a 3% difference in EMR, 9% in EUR and WPR, and 12% in SEAR. The largest decline in completeness of stool collection occurred in India, from 98% of AFP cases in January 2020 to 84% in April. The median number of days between the collection of the second stool specimen and receipt by the laboratory increased by 75% in AFR (from 4 to 7 days) and 33% SEAR and EMR (from 3 to 4 days) from 2019 to 2020. The mean number of days from the collection of the second stool to receipt by the laboratory increased by 70% from 2019 to 2020, from 5.4 to 9.2 days. The mean number of days between collection of the second stool specimen and receipt by the laboratory increased by 35% in EMR, from 4.6 in 2019 to 6.2 days in 2020, 47% in AFR, from 7.9 to 11.6 days, and 197% in SEAR, from 3.8 to 11.3 days, highlighting more occurrences of longer delays. The 197% increase in mean number of days between collection of second stool specimen and receipt by the laboratory in SEAR is primarily attributable to significant increases in India.

## Environmental Surveillance

During 2020, the mean number of monthly samples collected per active site declined from 1.2 in January to 0.8 in July, August, and September (33%) in AFR, from 2.3 in January to 0.8 in April and May (65%) in SEAR, and from 1.1 in January to 0.9 in March (18%) in EMR. Among 45 countries, 620 active environmental surveillance sites** reported to POLIS in 2020, an increase of 15% from the 537 sites that reported in 2019. Field staff members collected a mean of 1.6 samples per active site each month in 2019 compared with 1.1 per active site each month in 2020 (Table).

## Global Polio Laboratory Network

Countries reported movement and transportation restrictions that posed challenges with domestic or international transport of human and environmental specimens. At the height of these restrictions in June 2020, the inability to ship specimens to WHO-accredited laboratories led to the storage of over 850 human specimens (from AFP patients, AFP contacts, and healthy children) and approximately 50 environmental surveillance samples globally. With fewer AFP cases reported overall, GPLN tested 108,699 human specimens from January to September in 2020 compared with 167,056 human specimens during the same period in 2019, a 35% decline. Among regions for which data are available, environmental surveillance samples^††^ tested declined 30%, from 11,234 samples in 2019 compared with 7,853 in 2020 (Table).

## Discussion

Polio surveillance data indicate a 33% decline in AFP case reporting during the first 9 months of 2020 compared with the same period in 2019. Precautions taken to mitigate the spread of COVID-19 might have affected the ability of surveillance officers to conduct routine surveillance activities, which would have had an impact on the number of AFP cases reported. Despite a decline in case reporting, surveillance officers in most regions were able to collect two stool specimens from reported AFP patients with only a slight decrease in 2020, suggesting that the quality of case investigations did not decline. Assessment of completeness of collection of two stool specimens from patients with AFP by month found that the largest overall decline within the 9-month period occurred in India, from a high of 98% of AFP cases in January 2020 to 84% in April. The mean interval from the second stool collection to receipt by the laboratory increased 70%, from 5.4 to 9.2 days worldwide, indicating delays in stool transport. Although environmental surveillance has expanded in 2020, the mean number of samples collected per site declined, and transport of samples to the laboratory in AFR and SEAR was delayed. Several laboratories reported using polio staff members to support COVID-19 testing, which might have created a heavier workload for some staff members. Regional and country-specific variations in polio surveillance from 2019 to 2020 might have resulted from changes in COVID-19 epidemiology in some areas and associated restrictions on movement of polio staff members, diversion of resources from polio to the COVID-19 response, or the emergence and spread of type 2 circulating vaccine derived–poliovirus outbreaks (*4*).

Whereas the decline in polio surveillance coincided with the initial high spread of COVID-19, country-specific operational assessments would be required before attributing the declines to the pandemic. For instance, data from Pakistan suggest that the decrease in the number of reported AFP cases from 1,010 in March 2020 to only 585 in April corresponded with the increases in COVID-19 cases (16,117 COVID-19 cases by April 30) (*5*). In addition, in several countries, polio surveillance officers have played an important role in supporting the COVID-19 response, which affected the time they spent on polio surveillance activities (*6*). However, several instances of decreases in AFP reporting and environmental surveillance sample collection were not attributable to COVID-19. For example, a worker strike by polio field staff members in the Central African Republic in March 2020 resulted in a decline in AFP reporting; however, the number of reported AFP cases subsequently increased. In addition, a decrease observed in environmental surveillance collection in Angola in March and April 2020 was the result of challenges in transport that were unrelated to the pandemic and was not attributable to a decrease in sample collection (personal communication, Ticha Johnson Muluh, MD, World Health Organization, April 2020).

The findings in this report are subject to at least two limitations. First, although polio surveillance is often affected by many factors, including changes in resources and prioritized activities in outbreak-affected countries and neighboring countries, the amount and availability of funding, and global GPEI support for surveillance enhancement, none of these factors were included in this assessment. Second, surveillance trends before 2019 were not analyzed, restricting this analysis to monthly comparisons between 2020 and 2019.

The decline in AFP case reporting and sewage specimen collection, delays in transport, and limited surveillance activities suggest that global polio surveillance was negatively affected in 2020 by the COVID-19 pandemic. This has, in turn, negatively affected the ability of GPEI to detect poliovirus circulation. Recently, the impact of this was observed in delayed detection of poliovirus in Sudan, South Sudan, and Guinea caused by delays in shipping specimens. To mitigate further impact of the COVID-19 pandemic on polio surveillance, GPEI has implemented a series of measures to continue surveillance operations, including negotiating with national authorities for special specimen shipment clearance across closed borders, providing personal protective equipment for field officers, and updating guidance on polio surveillance practices in the context of COVID-19 (*3*). Surge staffing in countries with declines in polio surveillance performance could offset the diversion of resources to COVID-19. Implementing these measures will result in higher financial costs to polio field and laboratory surveillance operations and could affect sustainability. Although COVID-19 has introduced changes to routine operations that require new thinking and innovations, GPEI has a history of adapting to and addressing unforeseen challenges and remains committed to global polio eradication.

**Summary**

**What is already known about this topic?**

Surveillance for acute flaccid paralysis (AFP) is critical to detecting poliovirus circulation. Environmental (sewage) surveillance supplements AFP surveillance in many locations.

**What is added by this report?**

Poliovirus surveillance activities were modified as a result of the COVID-19 pandemic. Reported AFP cases declined 33% from January to September of 2020 compared with the same period in 2019, and the number of environmental samples per site declined. The decline in polio surveillance coincided with the spread of COVID-19.

**What are the implications for public health practice?**

Interruptions to poliovirus surveillance might have negative consequences on detection of poliovirus circulation. Continued analysis of AFP reporting trends is necessary to better understand the long-term impact to the eradication initiative . The Global Polio Eradication Initiative remains committed to global polio eradication.

## Footnotes

* Syndromic surveillance for polio identifies and tests acute flaccid paralysis that includes both polio and nonpolio.

† Two important indicators, the nonpolio AFP (NPAFP) rate and the percentage of stool specimens collected from AFP patients that are received in the lab in good condition, measure the sensitivity and quality of polio surveillance. rate is defined as the number of NPAFP cases per 100,000 children aged <15 years per year; an NPAFP rate ≥2 is considered sufficiently sensitive to detect circulating poliovirus. Stool adequacy is defined as the collection of adequate stool specimens from AFP patients (i.e., two stool specimens collected ≥24 hours apart and within 14 days of paralysis onset) and arrival of these specimens at a WHO-accredited laboratory by reverse cold chain (storing and transporting samples at recommended temperatures from the point of collection to the laboratory) and in good condition (i.e., without leakage or desiccation) from ≥80% of persons with AFP.

§
https://polioeradication.org/polio-today/polio-now/surveillance-indicators/the-global-polio-laboratory-network-gpln/ .

¶
https://extranet.who.int/polis.

** Active environmental sites: sites where at least one sample was collected and reported from November 1, 2019 to January 31, 2020.

†† Environmental surveillance data for AFR, SEAR, and EMR regions.
